# The Influence of Social Roles on the Well-Being of Older Caregivers: Social Isolation as a Potential Mediator

**DOI:** 10.1093/geroni/igaa057.119

**Published:** 2020-12-16

**Authors:** Sol Baik

**Affiliations:** University of Maryland, Baltimore, Baltimore, Maryland, United States

## Abstract

Engagement in productive roles has been associated with better health in later life. According to identity accumulation theory, more social roles help individuals be more socially integrated, leading to enhanced psychological well-being by diminishing the risk of focusing their energy into a single role. However, less is known about the mechanisms behind the relationship between productive roles and mental health of older caregivers. The aims of this study are to examine: (1) the association between productive social roles and psychological well-being for caregivers and non-caregivers, and (2) the mediating effect of social isolation on this relationship. The study analyzed 3,951 community-dwelling Medicare beneficiaries ages 65 or above from Rounds 1 to 3 of the National Health and Aging Trends Study (210 caregivers, 3,741 non-caregivers). Multiple-group analysis for structural equation modeling was conducted to compare caregivers to non-caregivers. For both caregivers and non-caregivers, having a volunteer role increased well-being, while paid employment was not significantly associated with well-being. Social isolation mediated neither employee nor volunteer role with well-being for both caregivers and non-caregivers. Assuming that volunteering for others without monetary compensation brings meaning in life, this finding supports the provision of services (e.g., respite care) that could supplement caregiving time, allowing caregivers to have more freedom to use the time in such community-engaging roles. Even though self-rated health was controlled, there is a chance that healthier individuals engage in productive roles. Thus, reciprocal relationships between productive roles and health should be considered in future investigations.

